# Predictive Potential of MALDI-TOF Analyses for Wine and Brewing Yeast

**DOI:** 10.3390/microorganisms10020265

**Published:** 2022-01-24

**Authors:** Junwen Zhang, Jeffrey E. Plowman, Bin Tian, Stefan Clerens, Stephen L. W. On

**Affiliations:** 1 Department of Wine, Food and Molecular Biosciences, Lincoln University, P.O. Box 85054, Lincoln 7674, New Zealand; Cherie.Zhang@lincolnuni.ac.nz (J.Z.); Bin.Tian@lincoln.ac.nz (B.T.); 2 AgResearch Ltd., Lincoln Research Centre, Lincoln 7674, New Zealand; Jeff.plowman@agresearch.co.nz (J.E.P.); Stefan.Clerens@agresearch.co.nz (S.C.); 3 Biomolecular Interaction Centre, University of Canterbury, Christchurch 8041, New Zealand; 4 Riddet Institute, Massey University, Palmerston North 4472, New Zealand; 5 Centre for Foods for Future Consumers, Lincoln University, P.O. Box 85054, Lincoln 7674, New Zealand

**Keywords:** MALDI-TOF analysis, commercial wine yeast, brewing yeast, winemaking, UMAP

## Abstract

The potential of MALDI-TOF profiling for predicting potential applications of yeast strains in the beverage sector was assessed. A panel of 59 commercial yeasts (47 wine and 12 brewing yeasts) was used to validate the concept whereby 2 culture media (YPD agar and YPD broth), as well as two mass ranges *m*/*z* 500–4000 and *m*/*z* 2000–20,000, were evaluated for the best fit. Three machine learning-based algorithms, PCA, MDS, and UMAP, in addition to a hierarchical clustering method, were employed. Profiles derived from broth cultures yielded more peaks, but these were less well-defined compared with those from agar cultures. Hierarchical clustering more clearly resolved different species and gave a broad overview of potential strain utility, but more nuanced insights were provided by MDS and UMAP analyses. PCA-based displays were less informative. The potential of MALDI-TOF proteomics in predicting the utility of yeast strains of commercial benefit is supported in this study, provided appropriate approaches are used for data generation and analysis.

## 1. Introduction

Wine is a complex product resulting from the interactions between yeasts and grape juice components, and each yeast strain within the same species has a specific impact on the final wine composition and sensory profile [1]. Diversity among commercial strains was highlighted through the unique phenotypic patterns of each strain [2]. The impact of the yeast on wine flavour is largely determined by the array of volatile substances (e.g., higher alcohols, acids, esters, carbonyls, and thiols) produced by the metabolism of grape juice components [3].

The adaptive divergence of genomics in response to different ecological niches allows the development of specific genetic groups of *Saccharomyces cerevisiae* in different fermented food (e.g., wine, beer, dairy products, and bread) and their natural habitats [4]. Along with the diverse fermentation environments, genotypes and phenotypes of *S. cerevisiae* are shaped via hybridization, polyploidization, pseudogenization, genome decay, gene duplication, and horizontal gene transfer to specifically adapt [5,6]. Commercial wine yeast strains are closely related, as demonstrated genetically by the microarray karyotyping analysis [7]; differences in the fermentation and organoleptic properties of each strain may arise from a small number of genetic changes. Most quantitative trait alleles exert considerable phenotypic variations among *S. cerevisiae* strains and alter conserved amino acid positions within protein-coding sequencing [8].

Matrix Assisted Laser Desorption/Ionization–Time-Of-Flight Mass Spectrometry (MALDI-TOF MS) is a novel proteomic approach, which has been widely applied in the identification and characterization of important microorganisms of food interest, such as pathogenic bacteria-*Listeria monocytogenes* [9], *Staphylococcus aureus* [10], *Thermophilic Campylobacter* [11], and non-typhoidal *Salmonella* [12]. In brief, MALDI-TOF MS is a technique based on “soft ionization”, where microbial cells are embedded in a suitable matrix that extracts and crystallises the native proteins and assists in their ionisation when exposed to a laser beam. The ions are then accelerated through an electrostatic field, and separated according to their *m*/*z* ratio until they reach the detector [13]. The resulting complex profile represents a species-specific fingerprint, conveying the ion mass (*m*/*z*) (typically *z* = 1) on the *x*-axis, and the number of ions of a particular size that hit the detector (peak intensity) on the *y*-axis.

The first time that MALDI-TOF MS was applied to the identification of yeasts (*S. cerevisiae* isolates) from fermented beverages was, to our knowledge, conducted by Vallejo et al. (2013) [14]. Recently, MALDI-TOF MS has been proven to be a powerful tool in wine yeast identification at species [15,16,17] and even strain levels [18,19]. Furthermore, Usbeck and Wilde [18] demonstrated the role of MALDI-TOF MS in revealing the relationship between wine yeast strains and their application potential, as well as comparable studies of brewing strains [20]. The underlying mechanism is the link between proteome and metabolism. Lafaye and Junot [21] showed that proteome and metabolic data could be correlated either positively or negatively depending on the growth conditions. Nonetheless, studies are few at this point, and none to our knowledge have combined investigations on both wine and brewing yeasts.

Machine learning is widely used to analyse complex data sets for prediction purposes [22,23]. Principal component analysis (PCA), Multidimensional scaling (MDS), and Uniform Manifold Approximation and Projection (UMAP) are three dimensionality reduction techniques (DRTs) for data visualization of machine learning-based methods [24]. PCA is a parametric linear projection that captures maximum variances in the dataset but is unable to capture the non-linear structures. MDS is the first non-parametric DRT that preserves topology and distances; it is able to capture non-linear structures but with limited capability [24]. UMAP is a new non-parametric approach put forward by McInnes and Healy [25] that builds on strong mathematical foundations and efficiently handles very large datasets.

To assess the use of MALDI-TOF analyses to predict potential applications of yeast in wine and beer production, we investigated several factors. First, the culture medium (YPD agar and broth) and mass range (*m*/*z* 500–4000 and *m*/*z* 2000–20,000) were evaluated for the best fit based on our previous work [17,26]. Thereafter, the three algorithms listed above, in addition to a classical hierarchical clustering approach, were adopted to investigate the potential of MALDI profiles in industrial yeast strains differentiation (commercial wine and brewing strains) and the potential application prediction. Furthermore, the manufacturer’s recommended application for each strain was incorporated to evaluate its potential in predicting strain utility for winemaking/beermaking.

## 2. Materials and Methods

### 2.1. Yeast Strains and Culture Conditions

A collection of 47 commercial wine yeast strains and 12 brewing yeast strains were tested (Table 1). Four additional type strains *S. cerevisiae* NCYC 505 ^T^, *S. paradoxus* NCYC 700 ^T^, *S. pastorianus* NCYC 396 ^T^, and *S. bayanus* NCYC 2578 ^T^ were purchased from NCYC (National Collection of Yeast Cultures, UK), and one isolate *S.cerevisiae* v128 was purified from Pinot Noir (PN) grape juice obtained from an organic winery, Greystone Wines, Waipara, New Zealand.

All commercial yeast strains were aseptically re-hydrated and inoculated into 15 mL YPD broth (Difco, c/o Thermo Fisher Scientific Ltd., Waltham, MA, USA) overnight at 28 °C. Afterward, the cultures were streaked onto YPD agar (Difco) and cultured under the same conditions for three days. Purified yeast strains were obtained and routinely stored at −80 °C in YPD glycerol stock (30%, *v*/*v*) after two-times subculture.

For MALDI-TOF MS analysis, yeast strains on YPD agar (Difco) were cultured for 72 h at 28 °C, whereas the strains in YPD broth (Difco) were cultured for 24 h at 28 °C.

### 2.2. MALDI-TOF MS

#### 2.2.1. Sample Preparation

The preparation of yeast strains harvested from YPD agar (Difco) was as described previously [17]. Samples from the liquid media were collected according to Usbeck and Kern [16]. In order to obtain enough yeast cells for MALDI analysis, 900 µL culture of YPD broth (Difco) was transferred into a 1.5 mL tube (Safe-Lock, Eppendorf, Hamburg, Germany) and centrifugated at 12,100× *g* for 4 min (Eppendorf AG, Minispin 5452, Hamburg, Germany). The supernatant was discarded, and the pellet was washed using 900 µL sterilized deionized water (produced by an ultra-pure water system by Barnstead GenPure Pro, Thermo Scientific, Waltham, MA, USA) 3 times. Subsequently, the pellet was resuspended into 300 µL deionized water, and vortexed for 1 min with 900 µL absolute ethanol (Fisher Chemical, Chicago, IL, USA). After centrifugation (12,100× *g*, 4 min), the pellet was air-dried in laminar-flow hood and stored at −20 °C prior to protein extraction.

To extract proteins, 50 µL of 70% formic acid (*v*/*v*) (Fisher Chemical, Chicago, IL, USA) was added to the yeast pellet and mixed thoroughly by vortexing for 1 min, then 50 µL of acetonitrile (ACN) (Fisher Chemical, Chicago, IL, USA) was mixed for the same time. Protein extract was obtained by centrifugation (12,100× *g*, 4 min). An equal volume of protein extract and α-cyano-4-hydroxycinnamic acid (HCCA) (Bruker Daltonics, Bremen, Germany) matrix solution (10 mg/mL in 75% ACN and 2.5% trifluoroacetic (TFA)) were mixed well, and 1 µL of this mixture was deposited onto the MALDI ground steel target plate (MTP 384, Bruker Daltonics, Billerica, MA, USA) until dry. For technical replication, each extract was spotted onto 3 individual wells, therefore yielding 9 spectra per strain.

#### 2.2.2. Mass Spectra Acquisition

MALDI-TOF mass spectra were automatically acquired on an Ultraflex III TOF/TOF MS instrument (Bruker Daltonics, Billerica, MA, USA), operating in positive ion detection mode using a SmartbeamTM laser at 200 Hz, pulsed-ion extraction time of 120 ns, and laser power 80%. The voltage of the ion source was set as 25.00 kV (ion source 1), 23.55 kV (ion source 2), and 6.01 kV (lens). Samples were analyzed using the linear detector at high mass range *m*/*z* 2000–20,000 and reflector detector at low mass range *m*/*z* 500–4000. The final spectrum was an average accumulation of 800 single spectra (low mass range *m*/*z* 500–4000) or 2000 single spectra (high mass range *m*/*z* 2000–20,000) gathered. Every single spectrum was recorded from 10 random raster spots.

The mass spectrometer was externally calibrated in every experiment at regular intervals, using the calibrant position in the middle of each tetrad of spots. For low mass range *m*/*z* 500–4000, peptide II standard (Bruker Daltonics, Billerica, MA, USA) (Bradykinin 1–7, [M + H]^+^ at *m*/*z* 757.3992, Angiotensin II, [M + H]^+^ at *m*/*z* 1046.5418, Angiotensin I, [M + H]^+^ at *m*/*z* 1296.6848, Substance P, [M + H]^+^ at *m*/*z* 1347.7354, Bombesin, [M + H]^+^ at *m*/*z* 1619.8223, ACTH clip 1–17, [M + H]^+^ at *m*/*z* 2093.0862, ACTH clip 18–39, [M + H]^+^ at *m*/*z* 2465.1983 and Somatostatin 28, [M + H]^+^ at *m*/*z* 3147.4710) was used. For high mass range *m*/*z* 2000–20,000, an in-house protein standard comprising Insulin, [M + H]^+^ at *m*/*z* 5734.52, Cytochrome C, [M + H]^+^ at 12,360.99 and [M + H]^2+^ at 6180.99, Myoglobin, [M + H]^+^ at 16,952.30 and [M + H]^2+^ at 8476.65), Aprotinin [M + H]^+^ *m*/*z* 6511.51, and β-lactoglobulin [M + H]^+^ *m*/*z* 18,363.00 was used.

#### 2.2.3. Data Analysis

Raw mass spectra were exported as .txt format using FlexAnalysis software (version 3.0. Bruker Daltonics, Billerica, MA, USA) and imported into software BioNumerics version 7.6 (Applied Maths, Kortrijk, Belgium). Spectra pre-processing was achieved at a default setting, but baseline subtraction with Rolling disc value was adjusted to 150. Kaiser Window value in smoothing and signal to noise ratio (S/N) in peak filtering were adjusted according to the quality of spectra.

A composite profile of each strain was obtained using 9 spectra derived from 3 technical replicates of each of 3 biological replicates. Cluster analysis was performed using the Pearson correlation coefficient and UPGMA (unweighted-pair group method with arithmetic mean) algorithm.

MDS and PCA analyses are available in BioNumerics version 7.6. MDS was performed based on a similarity matrix calculated using the metric algorithm Pearson coefficient. Pearson coefficient is insensitive to global differences in background and intensity as it contains an average intensity correction but is sensitive to local differences in intensity; thus, it is recommended for typing purposes and therefore adopted in our study [29]. PCA and UMAP were executed on peak classes detected by “peak matching” using the default settings (high mass: constant tolerance 1.9, linear tolerance 550 ppm, peak detection rate 10; low mass: constant tolerance 0.5, linear tolerance 300 ppm, peak detection rate 50). PCA was calculated with quantitative values (not just absent/present) and options to subtract the average character value over the characters. UMAP is founded on the assumptions that the data is uniformly distributed on the Riemannian manifold, the Riemannian metric is locally constant, and the manifold is locally connected, which was applied using the conda-forge packages for Python (Available online: https://umap-learn.readthedocs.io/en/latest/index.html (accessed on 19 January 2022)).

## 3. Results

### 3.1. MALDI-TOF Profiles of Strains Cultured on YPD Broth and YPD Agar

**Figure 1:**
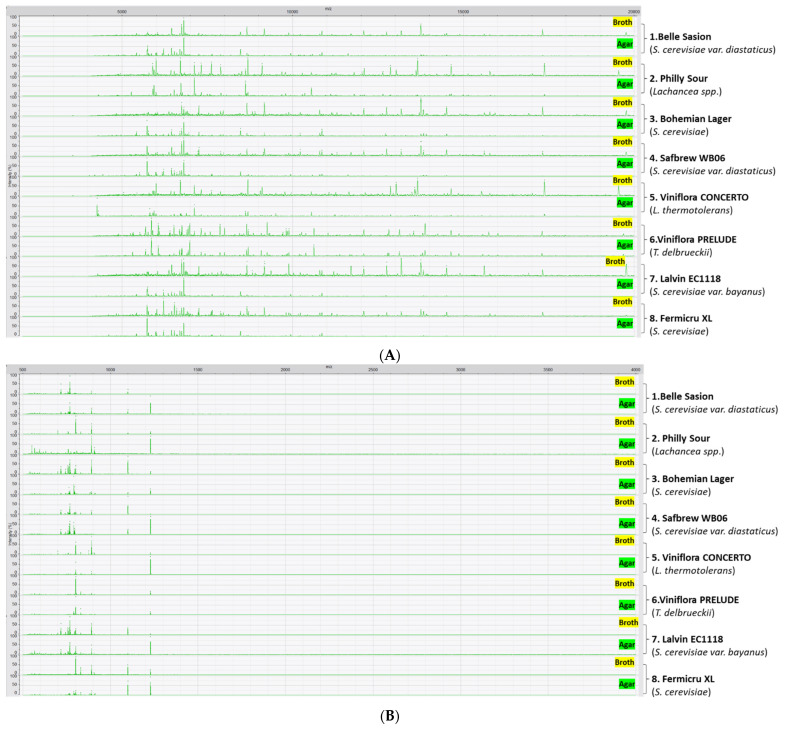
MALDI spectra of (**A**) high mass and (**B**) low mass of eight representative commercial strains cultured under YPD broth and YPD agar; 1–4: Brewing strains, 5–8: Wine strains.

Good quality MALDI profiles from each of the strains examined were obtained from cultures on each of the media used. Representative MALDI profiles of eight wine and brewing yeast strains are presented in Figure 1. Compared to strains grown on YPD agar, strains grown in YPD broth generated more peaks in a wider mass range, but the overall peak intensity was greatly decreased. Despite the visible differences of produced MALDI profiles, a set of common peaks with varying peak intensity (Low mass: *m*/*z* 712, 757, 767, 770, 891, 1100; High mass: *m*/*z* 5735, 5773, 6535, 6746, 6809, 7254, 7887, 8469, 8658, 10,219, 10,792, 10,854, 12,750, 13,750, 13,829, 14,506) were observed in samples from both growth media.

### 3.2. Strain Classification Using Cluster Analysis and Machine Learning Approaches

Although strain profiles produced from broth cultures contained more peaks, cluster (Figure S1) and machine learning-based analyses (Figure S2) tended to correlate poorly with extant information concerning the utility of individual strains. These results are not considered further.

Cluster analysis of all the *S. cerevisiae* strains (winemaking and brewing) exhibited different grouping based on their high-, low- and combined-mass spectra profiles (Figure S3). With a thorough visual examination of the spectra patterns, 95% and 85% were indicated as the threshold values in high mass and low mass dendrograms, respectively, resulting in 17 and 20 subclusters. Likewise, 18 subclusters were recognized in the high-low combined dendrogram when 85% was set as the threshold value. Compared to high mass clustering, the industrial strains differentiation was better illustrated by low mass profiles where all the brewing strains were clustered together (group 12–20). In either the high or low mass dendrogram, strains of Velluto Evolution, Fermi champ, Renaissance Vivace, Belgian Wit, Belle Saison, Verdant IPA, NWS Ale, LalBrew Köln, and BRY97_American were affiliated. Three Lager strains of Californian Lager, Bohemian Lager, and Saflager 23 clustered together in the low mass dendrogram analysis, while the former two strains were mixed with wine strains (Group 2) in the high mass dendrogram. Strains recommended for Champagne production (PDM) fell into three subclusters in both dendrograms, containing four different strains of *S. cerevisiae*, *S. cerevisiae* var. *cerevisiae*, *S. cerevisiae* × *S. cariocanus*, and *S. cerevisiae* var. *bayanus*.

**Figure 2:**
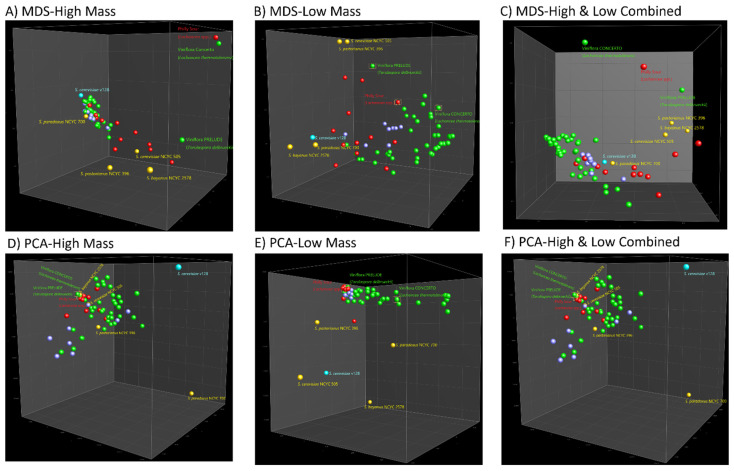
MDS analysis of (**A**) high mass; (**B**) low mass and (**C**) high and low combined data, PCA analysis of (**D**) high mass and (**E**) low mass, and (**F**) high and low combined data of 62 yeast strains-45 wine strains (green/purple), 12 brewing strains (red), *S. cerevisiae* v128 (blue), *S. cerevisiae* NCYC 505 (yellow), *S. paradoxus* NCYC 700(yellow), *S. pastorianus* NCYC 396 (yellow), *S. bayanus* NCYC 2578 (yellow).

Representation of inter-strain relationships among all strains examined using each of the multidimensional scaling techniques (MDS, PCA, and UMAP) was generally more nuanced. The PCA plot gave the poorest degree of association between strain utility and even species identity, with the most obvious outliers to be the major group represented by a local vineyard isolate of *S. cerevisiae*, and the type strain of *S. paradoxus* NCYC 700 (Figure 2D). The UMAP analysis distributed most of the *S. cerevisiae* strains recommended for winemaking among five groups, although some of these contained strains recommended for beer and Champagne production (PDM) as well (Figure S4A). The MDS plot displayed a more consistent grouping of strains with better alignment of their recommended use and taxonomic relationship. Brewing-related strains (*S. cerevisiae* NCYC 505, *S. bayanus* NCYC 2578, and *S. pastorianus* NCYC 396) were aligned with the commercial brewing group (red dots), whereas *S. cerevisiae* v128 (indigenous yeast isolate) appeared close to, but distinct from, wine and PDM group strains, and quite close to the *S. paradoxus* type strain (Figure 2A). Strains recommended for Champagne production (PDM) were somewhat at an interface between the wine and beer producers.

### 3.3. Separate Analyses Were Undertaken on S. cerevisiae Strains for Which Recommendations Were Extant for Particular Wine Styles

**Figure 3:**
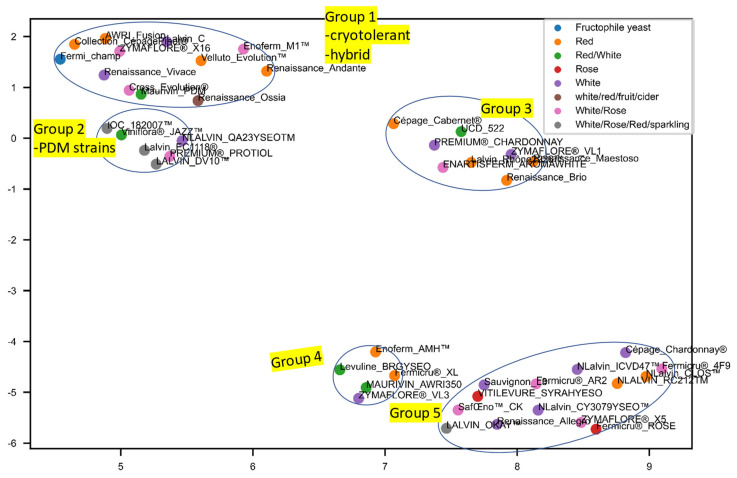
UMAP analysis of high mass profiles of 45 commercial wine *Saccharomyces* strains.

The 45 *Saccharomyces* wine yeast strains we selected to cover a wide range of applications, which can be roughly divided into 9 categories, namely, for the production of white wine, red wine, red and white wine, white/rose/red wine, rose wine, white and rose wine, white/red/fruit/cider, white/rose/red/sparkling wine, and one fructophile yeast Fermicru Champ used for tackling stuck fermentation. MDS and PCA did not show appreciable groupings based on their purposes in winemaking for different wine styles (Figure S5). However, UMAP distinguished five groups containing strains with some agreement where winemaking style recommendations were taken into account (Figure 3 and Figure S6). Group 1 was dominated by strains recommended for red wine production. Group 2 contained the majority of strains used to produce PDM and was classified as *S. cerevisiae* var. *bayanus*. Compared to the other three groups of strains, these two groups seem to have a stronger tolerance to low fermentation temperature and high alcohol content according to the manufacturing information, and their overall peak intensity and peak numbers were relatively low (Figure S7). Groups 3 and 4 are also well-populated with strains for red winemaking, and rosé too, in the case of Group 3. Group 5 contains mainly white wine yeast strains, mostly recommended for producing Sauvignon Blanc and Chardonnay wines.

**Figure 4:**
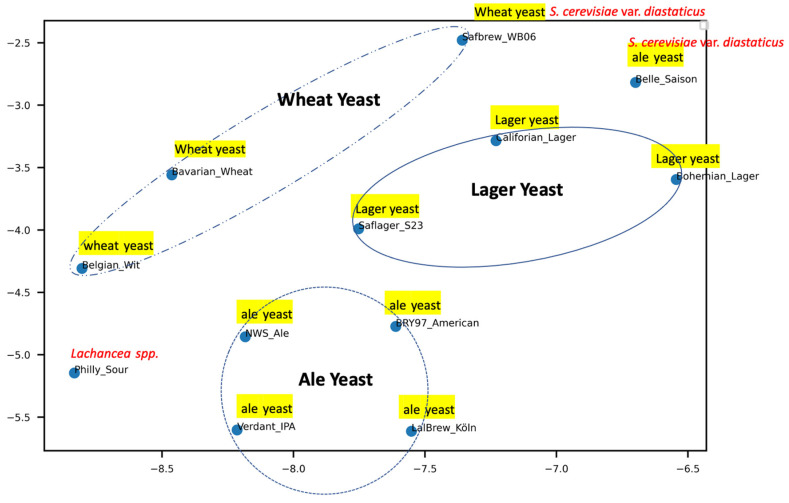
UMAP analysis of high mass profiles of 12 commercial brewing strains.

Although only 12 brewing strains were examined, strains belonging to wheat, lager, and ale were grouped separately, in particular, when the high mass was analysed (Figure 4 and Figure S8). The outlier ale yeast Belle Saison and wheat yeast Safbrew_WB06 were placed closer as their identity as *S. cerevisiae* var. *diastaticus*. The single strain representing the non-*Saccharomyces* species (*Lachanchea* spp.) on the left bottom is suggested to produce a sour beer.

## 4. Discussion

The interaction between yeast strain and grape varietal is integral to the flavour profile of the wine. During fermentation, the performance of each yeast strain is affected by the grape must composition, as well as the fermentation conditions. Therefore, the strain may not perform as expected if the growth condition (e.g., matrice and temperature) is not compatible with the expression of desired characters [30]. Some strains can produce metabolites that enhance mouthfeel (e.g., Lalvin ICV D47 and Lalvin CLOS), modify varietal aroma through enzymatical and chemical cleavage of aroma precursors (e.g., Lalvin QA 23 with high β-glucosidase activity), and improve the wine stability by increasing yeast mannoproteins [30]. Therefore, it is important to choose an appropriate yeast strain for making wine from a particular grape variety. We further examined the prospects of identifying strain utility for fermentation processes using proteome characterization by MALDI-TOF MS.

Based on optimized parameters described previously [17,26], YPD agar and YPD broth were selected as the culture media in this work. Although differences were observed among MALDI profiles, a set of core peaks remained constant, which was consistent with the reports from Usbeck and Kern [16], Reich and Bosshard [31], and Moothoo-Padayachie and Kandappa [19], who also stated that the variations did not compromise the accurate identification on species/strain level. The common peaks are likely to be the ribosomal or housekeeping proteins, whose expression is vital to the basic cellular function irrespective of the growth conditions. Approximately half of the peaks in the MALDI spectra could be assigned to such highly abundant ribosomal proteins, with some peaks matched to post-translationally modified ribosomal proteins [32].

Wine yeast strains are genomically and phenotypically distinct from other industrial yeast strains (beer, bread, and sake), as well as laboratory strains, pathogenic strains, and ‘wild’ yeast strains [33]. Dunn and Richter [28] pointed out that *NFT*1, *FLO*1, *AAD*6, and *AGP*3 genes present in most wine yeast strains but absent in most non-wine yeast strains, are important marker genes to differentiate yeast strains based on their application. Likewise, MALDI profiles successfully differentiated the wine and brewing yeast strains tested in this work. The domestication of diverse industrial *S. cerevisiae* populations (e.g., wine, beer, and bread) has been achieved through long-term evolution under selective pressures of various sources, like ancient customs, human migration, and industrial practice, encouraging the development of customized genomes for better adaption in new ecological niches [4,34,35]. In addition, species *S. paradoxus*, *S. bayanus*, and *S. pastorianus* are also of industrial importance in food fermentation, as well as their interspecific/intraspecific hybrids [36]. *S. paradoxus* is commonly found on the exudates and bark of deciduous trees [37]. In wild environments, *S. paradoxus* rarely cross-fertilizes with *S. cerevisiae*, but conditions in the intestine of some insects favour their hybridization, potentially creating an adaptive environment [38]. Lager beer yeast *S. pastorianus*, especially amenable to cooler fermentation temperature, is a naturally occurring interspecies hybrid of *S. cerevisiae* and *S. eubayanus* [34]. Type strain *S. bayanus* NCYC 2578 is a hybrid between *S. eubayanus* and *S. uvarum* [39]. Their genetic structure is reflected in our MALDI-TOF analysis, whereby *S. pastorianus* NCYC 396 and *S. cerevisiae* NCYC 505 are closer than NCYC 396 and NCYC 2578 in both high-(44.6% vs. 40.8%) and low-(74.1% vs. 9.8%) mass spectra. Moreover, their proximity to the brewing group of strains not only exhibited the capacity of this methodology as a powerful identification tool, but also showed the potential of MALDI-TOF MS as a predictive phenotypic tool.

Data interpretation is greatly affected by the algorithm used [16]. Dimensionality reduction techniques (DRTs) can provide an in-depth insight into subgrouping with an intuitive data interpretation. In this study, MDS was calculated based on the similarity matrix based on the Pearson coefficient, and then each data point was assigned using a non-linear least-squares fit, minimizing the distances between the data points [23]. MDS appears to be a valuable alternative to the traditional clustering methods. In our study, PCA was the least informative of the DRT methods applied, yielding the poorest correlation of strain grouping with industry recommendation, although it is one of the oldest and best-known DRTs. However, with the help of UMAP, 45 *Saccharomyces* commercial strains were classified into 5 groups using the high mass profiles, where MDS and PCA failed. It could be due to the fact that UMAP allows a more accurate representation of local trends, while PCA is better at the visualization of global data structure [24].

Low mass profiles allow for a rough classification of the industrial strains under MDS analysis (Figure 2B), but its combination did not significantly enhance the differential capacity of high mass profiles (*m*/*z* 2000–20,000). PCA and UMAP could not extract meaningful information from the limited peak classes (7 peak classes) as well. Interestingly, the data comparison between the UPGMA-based high- and low- dendrogram substantiated the potential of low-mass data as a powerful biotyping tool. The grouping of certain strains in two dendrograms was observed to be consistent. Velluto Evolution, the only hybrid of *S. cerevisiae*/*uvarum*, was in a single branch in both dendrograms. A similar case applies to Fermi champ, a special strain for tackling stuck fermentation, which is claimed to be *S. cerevisiae* (ex *bayanus*) but separated from the other strains of *S. cerevisiae* (ex *bayanus*). It is reasonable to infer that the MALDI profile clustering is an interaction between the genetic and phenotypic traits of individual strains. Overall, low mass profiles allowed a more detailed strain classification but were also affected by the phenotypes. In accordance with our previous inference, the low mass profiles did contribute to the added benefits of amplifying the intraspecific features [17].

When looking at the UMAP subgroups, Group 1 was dominated by the hybrid strains, which usually combine and exhibit superior phenotypic qualities over parent strains. Yeasts belonging to species of *S. bayanus* (*S. uvarum* × *S. eubayanus*)/*S. uvarum* are usually related to the ability to ferment at lower temperatures and greater production of aroma-active higher alcohols [40]. For example, the natural intraspecific hybrid Cross Evolution (*S. cerevisiae* var. *cerevisiae*) is ideal for white and rosé wines with high aromatic intensity (including ester production) and low fermentation temperature, and the interspecific hybrid Velluto Evolution (*S. cerevisiae* × *S. uvarum*) is characterized by high production of glycerol, phenyl ethanol generation, and good tolerance to low fermentation temperature (e.g., at 12 °C). Group 2 was represented by the PDM strains with two non-PDM strains of Premium Protiol and Viniflora Jazz. The collection of PDM strains is a special group from wine yeasts mainly described as *S. cerevisiae* var. *bayanus* [41], which is considered to be an intermediate group between non-wine and wine strains [28]. A related observation using the MDS and UMAP algorithms is that the PDM group is distributed at the interface between wine and brewing strains.

*S. cerevisiae* var. *bayanus* is a variety of *S. cerevisiae* that was reduced from its former species status (*S. bayanus*), as it could only be differentiated from *S. cerevisiae* by the fermentation of galactose [41]. The almost identical genotypes of the majority of the PDM group suggested that they may have arisen from a single progenitor strain or a highly interrelated progenitor population [27]. Coi and Bigey [42] inferred that the PDM group (Champagne related strains) might result from the cross between flor and wines gene pool, which benefits from the ability of flor strains under poor nutritional conditions and ethanol stress during the second fermentation of the “*Prise de mousse*” step that imposes a second anaerobic growth. In this sense, it explains its location as a neighbour beside the hybrid Group 1, having an overall stronger tolerance to low fermentation temperature and high alcohol content, as well as the fructophile strain Fermicru Champ for tackling stuck fermentation. Zymaflore VL3 in Group 4, Zymaflore X5, and Fermicru 4F9 in Group 5 are representative “thiol-releasing” wine yeasts suitable for the full aroma potential development of Sauvignon Blanc wine [43]. Similarly, Zymaflore X5 and Fermicru 4F9 were also shown to be a closer relationship in the study of Hart and Jolly [43].

As stated by the manufacturer, Premium Protiol is a strain of *S. cerevisiae*, but Silhavy-Richter, Hack [40] inferred it could be an unidentified interspecific hybrid of *S. cerevisiae* and *S. bayanus*, and strain QA 23 could be a derivative of EC 1118. Microsatellite analysis is not affected by physiological parameters, whereby the two PDM strains QA 23 and EC118, as well as the non-PDM strain Premium Protiol, were clustered together as our MALDI result indicated [40]. In addition to the natural hybridization between *Saccharomyces* strains, gene transfer between *Saccharomyces* and non-*Saccharomyces* species was observed in strain EC1118, the major wine contaminant *Zygosaccharomyces bailii* was identified as one donor species [44]. Additionally, aroma compounds produced were shown to be temperature dependent and vary between pure strain and hybrids; the best aroma producers at 28 °C were *S. cerevisiae* strains, whereas *S. uvarum* and some hybrids excelled at 12 °C [45]. It may corroborate our observation that an overall lower peak numbers and intensity (low protein expression) of Group 1 and 2 strains (most of the hybrids) under YPD agar (28 °C) was seen compared to the other 3 groups.

As discussed above, specific MALDI profiles obtained from yeasts grown on the YPD agar at 28 °C cannot reflect the real-time protein expression of yeast strains under winemaking conditions. Unlike the complex composition in grape must, YPD agar is a defined medium comprising four components (yeast extract, sugar, peptone, and agar). A previous study indicates different metabolites detected by MALDI-TOF analysis when winemaking yeast is cultured in these different conditions [26]. Nonetheless, the use of defined media for MALDI-TOF characterization of winemaking yeast is still recommended, based on the clarity of the spectra obtained and general support of yeast growth in comparable conditions [26]. The release of aroma compounds is strongly linked to the presence of aroma precursors in fermenting media [46]. The wine aromatic profiles can be modulated by employing different yeast species/strains and fermentation temperatures [45,46,47]. For example, according to the manufacturer instructions, strain EnartisFerm Aroma White is recommended for the thiolic varieties such as Sauvignon Blanc and Pinot Blanc with more citrus and mineral notes produced at 14–16 °C and more aromas of tropical white fruit produced at 17–20 °C. Enoferm AMH is a colour-friendly yeast and particularly suited for Pinot Noir and Zinfandel, partially due to its low levels of enzyme production responsible for colour loss and its long lag phase plus low-medium fermentation rate also allows the expression of indigenous microflora. In the face of fluctuating environments, limitations in gene expression play a role in phenotypic diversity at the expense of growth rates [48]. The early study of Batistote and da Cruz [49] suggested that the sugar types and concentration, the nitrogen source complexity, and the yeast genetic background collectively influenced the optimal industrial yeast fermentation performance. Moreover, the biotechnological application of yeast strains is often contradictory. According to the instruction, UCD522 (Group 3, red wine yeast group) is recommended for white and red wines, and more popular for red wines. However, Carrau and Medina [50] suggest it is more suitable for fermentation of neutral varieties. The data presented in this study corroborates the study of Usbeck et al. (2014) [18] in indicating a role for rapid and cost-effective MALDI-TOF profiling to predict the potential of individual yeast strain for production of specific or distinct wine varietals. However, to better correlate the relationship between the MALDI data and the oenological traits of wine yeast strains, a more complete and objective analysis of metabolites produced is required.

## 5. Conclusions

In conclusion, MALDI profiles generated under YPD agar have a better performance for the purpose of industrial strains differentiation than YPD broth. Neither MDS nor PCA analysis could group wine strains according to their recommended application in winemaking. However, UMAP provided the predictive potential in clustering strains of similar functionality and/or organoleptic attribute. In summary, further studies and subsequent algorithm exploration and data mining are warranted to fully evaluate the relationship of the MALDI profile to practical application in wine production. MALDI-TOF MS is worth continuing investigation as a powerful tool for yeast strain application prediction to simplify and expedite the selection of relevant indigenous wine yeasts for the development of new and interesting wine styles from an entirely natural base.

## Figures and Tables

**Table 1:** A total of 47 commercial wine yeast strains and 12 commercial brewing yeasts strains used in this work, in which wine strains were kindly provided by Lincoln University Winery, and brewing strains were purchased from BREWSHOP.

Commercial Strains	Genetic Background
Wine strains	
AWRI Fusion *	*S. cerevisiae* × *S. cariocanus*
Cepage Cabernet	*S. cerevisiae*
Cepage Chardonnay	*S. cerevisiae*-Strain n° LW05
Collection Cepage Pinot	*S. cerevisiae*
Cross Evolution	*S. cerevisiae* var. *cerevisiae*
Enartisferm Aroma White	*S. cerevisiae*
Enoferm AMH™	*S. cerevisiae* var. *cerevisiae*
Enoferm M1	*S. cerevisiae*
Fermi champ	*S. cerevisiae* (ex *bayanus*)
Fermicru 4F9 *	*S. cerevisiae*-Strain n° 4F9
Fermicru AR2	*S. cerevisiae*-Strain n° L0122
Fermicru Rose *	*S. cerevisiae*-Strain n°LW10
Fermicru XL	*S. cerevisiae*-Strain n° CECTA 11947
IOC 18-2007 *	*S. cerevisiae* var. *bayanus*
Lalvin C	*S. cerevisiae* var. *bayanus*
Lalvin CLOS	*S. cerevisiae* var. *cerevisiae*
Lalvin CY 3079	*S. cerevisiae* var. *cerevisiae*
Lalvin DV10 *	*S. cerevisiae* var. *bayanus*
Lalvin EC1118 *	*S. cerevisiae* var. *bayanus*
Lalvin ICV D47	*S. cerevisiae* var. *cerevisiae*
Lalvin OKAY	*S. cerevisiae* var. *cerevisiae*
Lalvin RC212	*S. cerevisiae* var. *cerevisiae*
Lalvin Rhone 2226	*S. cerevisiae*
LalvinQA 23 *	*S. cerevisiae*
Levuline BRG	*S. cerevisiae*
Maurivin AWRI 350	*S. cerevisiae*
Maurivin PDM *	*S. cerevisiae* (var. *bayanus*)
Premium Chardonnay	*S. cerevisiae*
PREMIUM^®^ PROTIOL	*S. cerevisiae*
Renaissance Allegro	*S. cerevisiae bayanus*
Renaissance Andante	*S. cerevisiae*
Renaissance Brio (Brioso)	*S. cerevisiae*
Renaissance Maestoso	*S. cerevisiae*
Renaissance Vivace	*S. cerevisiae bayanus*
Rennaissance Ossia	*S. cerevisiae*
Safoeno^TM^ CK	*S. cerevisiae*
Sauvignon L3	*S. cerevisiae*
UCD522	*S. cerevisiae*
Velluto Evolution™	*S. cerevisiae*/*uvarum*
Viniflora Jazz	*S. cerevisiae*
Viniflora^®^ PRELUDE™	*Torulaspora delbrueckii*
Viniflora^®^ CONCERTO™	*Lachancea thermotolerans*
Vitilevure Syrah	*S. cerevisiae*
Zymaflore VL1	*S. cerevisiae*
Zymaflore VL3	*S. cerevisiae*
Zymaflore X5	*S. cerevisiae*
ZYMAFLORE^®^ X16	*S. cerevisiae*
Brewing strains	
BRY-97 American West Coast Yeast	*S. cerevisiae*
LalBrew KÖln	*S. cerevisiae*
Belle Saison	*S. cerevisiae* var. *diastaticus*
Mangrove Jack’s New World Strong Ale Yeast	*S. cerevisiae*
Philly Sour	*Lachancea* spp.
LalBrew Verdant IPA	*S. cerevisiae*
Mangrove Jack’s Californian Lager Yeast	*S. cerevisiae*
Mangrove Jack’s Bohemian Lager Yeast	*S. cerevisiae*
Saflager S-23 Yeast	*S. cerevisiae*
Mangrove Jack’s Bavarian Wheat Yeast	*S. cerevisiae*
Mangrove Jack’s Belgian Wit Yeast	*S. cerevisiae*
Safbrew WB-06 Wheat Yeast	*S. cerevisiae* var. *diastaticus*

* Fermicru_ROSE, AWRI_Fusion, Lalvin DV10, Fermicru 4F9, Lalvin EC 1118, Lalvin QA 23, IOC 18-2007, and Maurivin PDM are associated with the *Prise de Mousse* (PDM) collection of Champagne production [27,28].

## Data Availability

The MALDI-TOF spectra for strains examined are held in an in-house database not intended for further dissemination.

## Supplementary Materials

The following supporting information can be downloaded at: https://www.mdpi.com/article/10.3390/microorganisms10020265/s1, Figure S1: Cluster analysis of high mass profiles of 59 commercial strains (47 wine and 12 brewing strains) grown under (A) YPD agar and (B) YPD broth.; Figure S2: (A) MDS analysis and (B) PCA analysis of high mass, low mass and high-low combined of 59 commercial yeast strains (47 wine and 12 brewing strains) under YPD broth and YPD agar; Figure S3: Cluster analysis of high mass profiles of 59 commercial strains (47 wine and 12 brewing strains) grown on YPD agar (A) High Mass, (B) Low Mass and (C) High & Low Combined; Figure S4: UMAP analysis of (A) high mass, (B) low mass and (C) high & low combined data of 62 yeast strains-45 wine strains (green/purple), 12 brewing strains (red), *S. cerevisiae* v128 (blue), *S. cerevisiae* NYC 505 (yellow), *S. paradoxus* NCYC 700(yellow), *S. pastorianus* NCYC 396 (yellow), *S. bayanus* NCYC 2578 (yellow); Figure S5: MDS and PCA analysis of 45 commercial wine *Saccharomyces* strains; Figure S6: UMAP analysis of (A) Low mass and (B) High & Low combined data of 45 commercial wine *Saccharomyces* strains; Figure S7: Heatmap of peak classes detected from 45 commercial wine strains and grouped according to UMAP analysis. Red colour represents the highest peak intensity, whereas the blue colour represents the lowest peak intensity; Figure S8: UMAP analysis of (A) Low mass and (B) High & Low combined data of 12 commercial brewing strains.
