# Supplementary material for: Predictive Potential of MALDI-TOF Analyses for Wine and Brewing Yeast
Source: Microorganisms. 2022 Jan 24;10(2):265. doi: 10.3390/microorganisms10020265 (PMC8875952; doi:10.3390/microorganisms10020265)
Supplement: Supplementary file 1 [file microorganisms-10-00265-s001.zip › microorganisms-1525621-SI.pdf]

Supplementary Materials

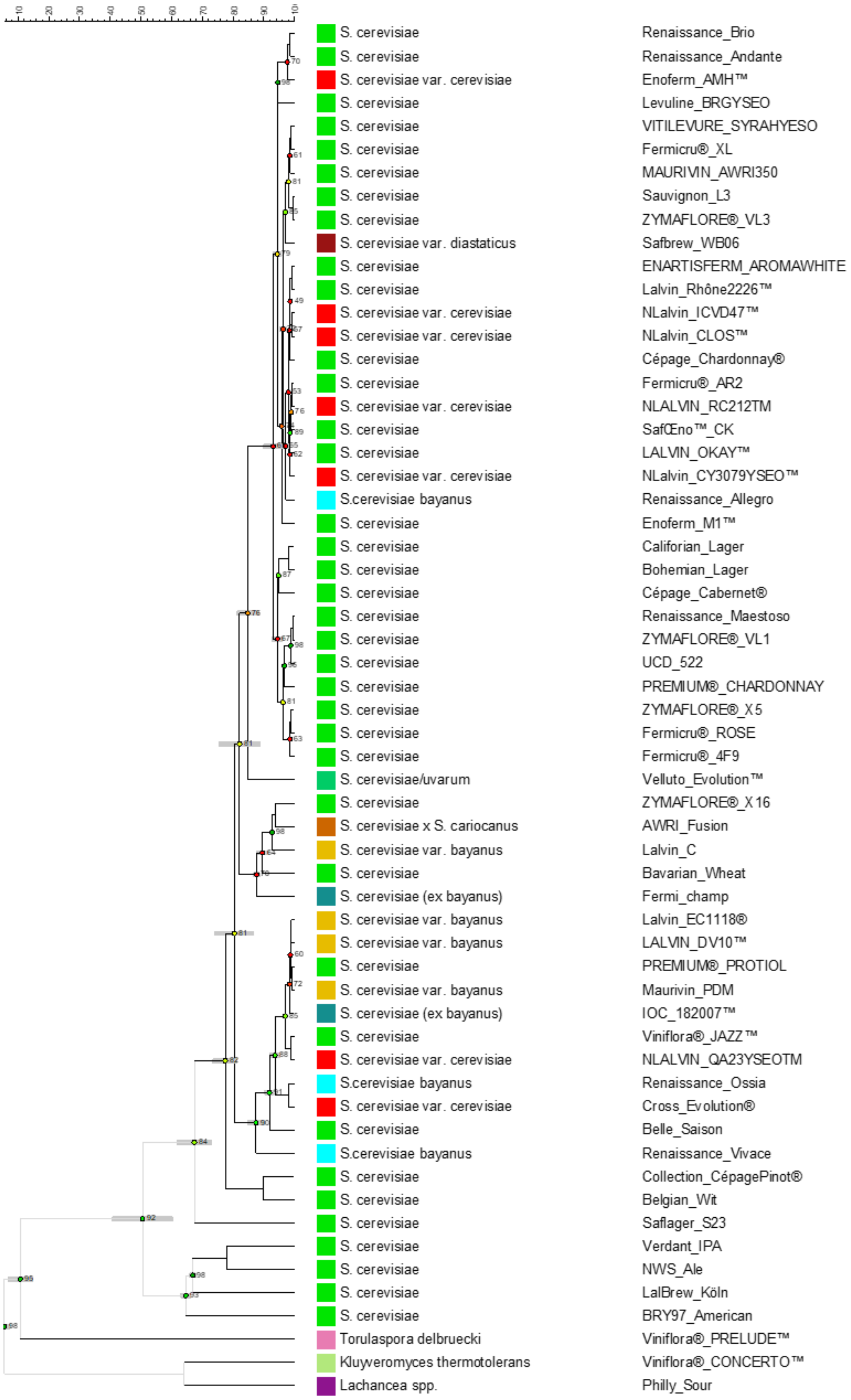

(A)

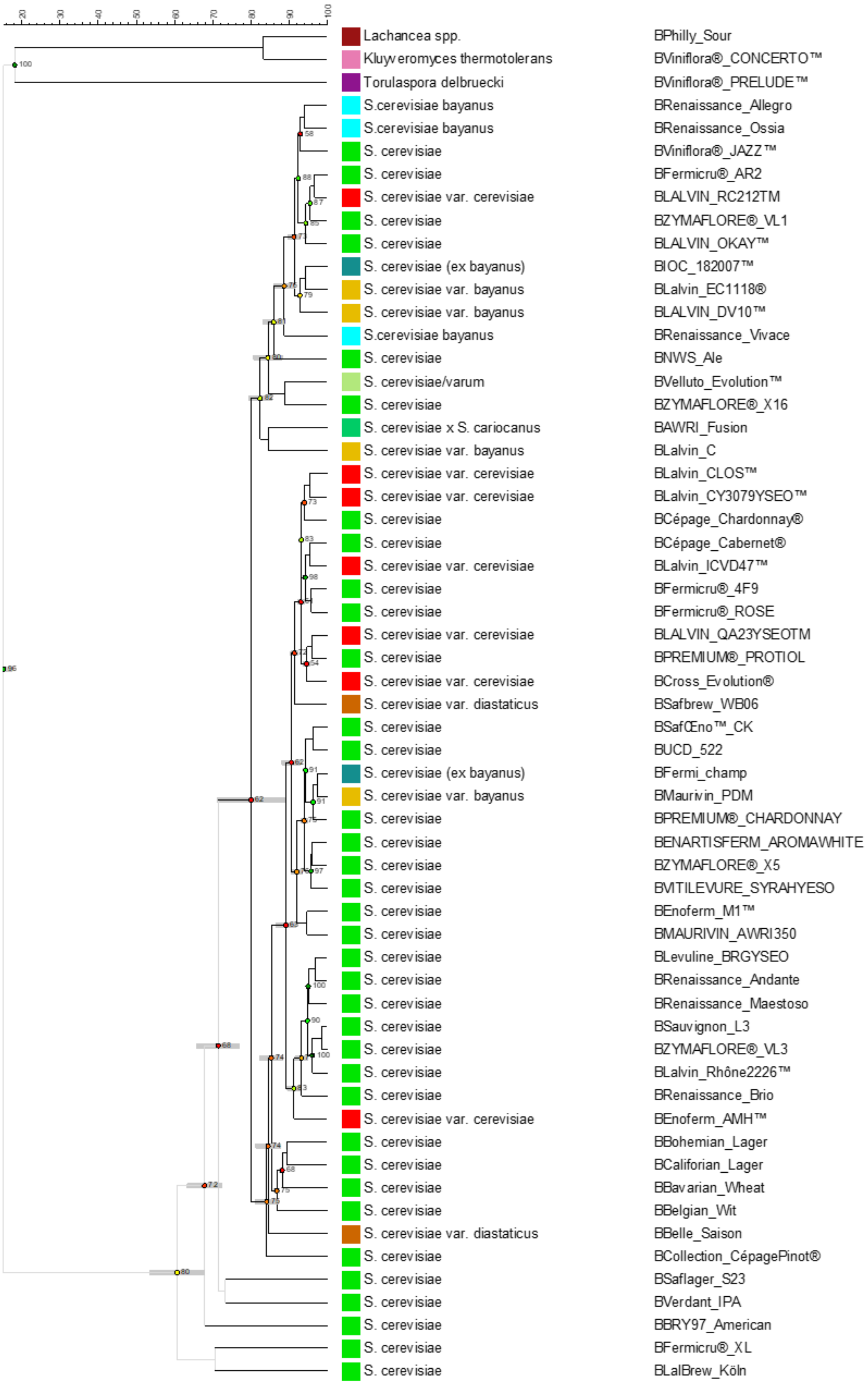

(**B**)

Figure S1. Cluster analysis of high mass profiles of 59 commercial strains (47 wine and 12 brewing strains) grown under (**A**) YPD agar and (**B**) YPD broth.

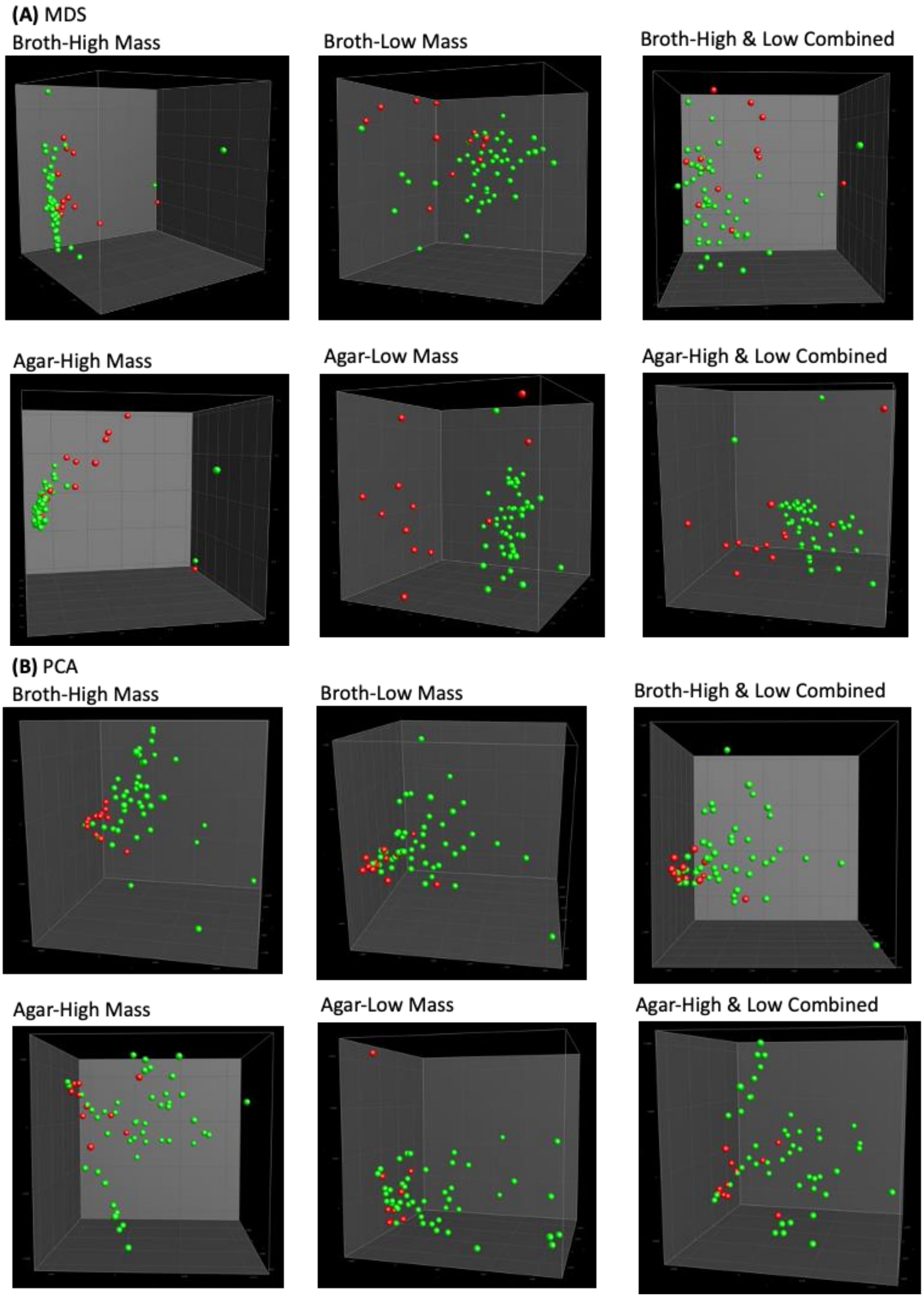

Figure S2. (**A**) MDS analysis and (**B**) PCA analysis of high mass, low mass and high-low combined of 59 commercial yeast strains (47 wine and 12 brewing strains) under YPD broth and YPD agar.

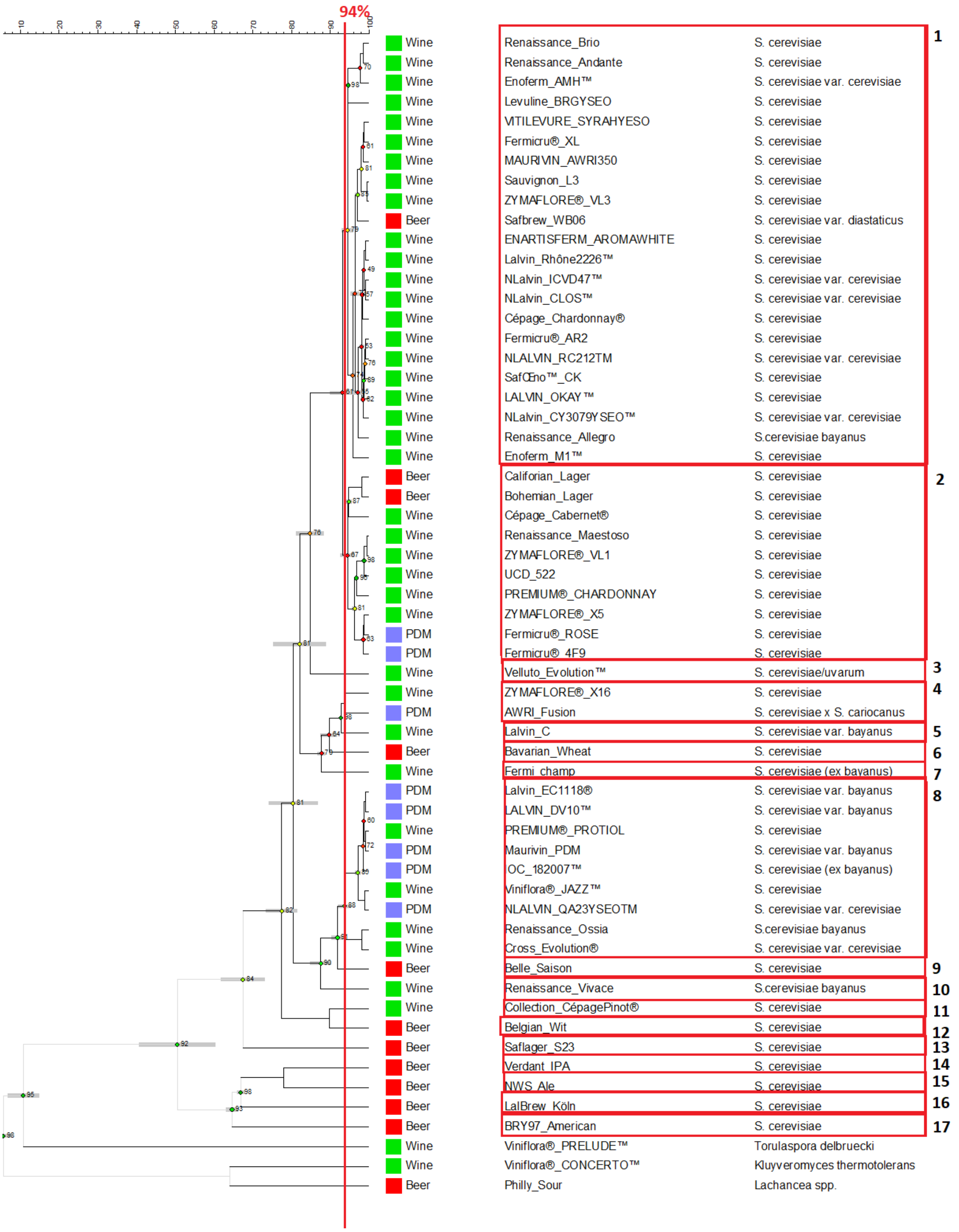

94%
10 20 30 40 50 60 70 80 90 100
Wine Renaissance_Brio S. cerevisiae
Wine Renaissance_Andante S. cerevisiae
Wine Enoferm_AMH™ S. cerevisiae var. cerevisiae
Wine Levuline_BRGYSEO S. cerevisiae
Wine VITILEVURE_SYRAHYESO S. cerevisiae
Wine Fermicru®_XL S. cerevisiae
Wine MAURIVIN_AWRI350 S. cerevisiae
Wine Sauvignon_L3 S. cerevisiae
Wine ZYMAFLORE®_VL3 S. cerevisiae
Beer Safbrew_WB06 S. cerevisiae var. diastaticus
Wine ENARTISFERM_AROMAWHITE S. cerevisiae
Wine Lalvin_Rhône2226™ S. cerevisiae
Wine NLalvin_ICVD47™ S. cerevisiae var. cerevisiae
Wine NLalvin_CLOS™ S. cerevisiae var. cerevisiae
Wine Cépage_Chardonnay® S. cerevisiae
Wine Fermicru®_AR2 S. cerevisiae
Wine NLALVIN_RC212TM S. cerevisiae var. cerevisiae
Wine SafŒno™_CK S. cerevisiae
Wine LALVIN_OKAY™ S. cerevisiae
Wine NLalvin_CY3079YSEO™ S. cerevisiae var. cerevisiae
Wine Renaissance_Allegro S.cerevisiae bayanus
Wine Enoferm_M1™ S. cerevisiae
1
Beer Califorian_Lager S. cerevisiae
Beer Bohemian_Lager S. cerevisiae
Wine Cépage_Cabernet® S. cerevisiae
Wine Renaissance_Maestoso S. cerevisiae
Wine ZYMAFLORE®_VL1 S. cerevisiae
Wine UCD_522 S. cerevisiae
Wine PREMIUM®_CHARDONNAY S. cerevisiae
Wine ZYMAFLORE®_X5 S. cerevisiae
PDM Fermicru®_ROSE S. cerevisiae
PDM Fermicru®_4F9 S. cerevisiae
2
Wine Velluto_Evolution™ S. cerevisiae/uvarum
3
Wine ZYMAFLORE®_X16 S. cerevisiae
PDM AWRI_Fusion S. cerevisiae x S. cariocanus
4
Wine Lalvin_C S. cerevisiae var. bayanus
5
Beer Bavarian_Wheat S. cerevisiae
6
Wine Fermi_champ S. cerevisiae (ex bayanus)
7
PDM Lalvin_EC1118® S. cerevisiae var. bayanus
PDM LALVIN_DV10™ S. cerevisiae var. bayanus
Wine PREMIUM®_PROTIOL S. cerevisiae
PDM Maurivin_PDM S. cerevisiae var. bayanus
PDM OC_182007™ S. cerevisiae (ex bayanus)
Wine Viniflora®_JAZZ™ S. cerevisiae
PDM NLALVIN_QA23YSEOTM S. cerevisiae var. cerevisiae
Wine Renaissance_Ossia S.cerevisiae bayanus
Wine Cross_Evolution® S. cerevisiae var. cerevisiae
8
Beer Belle_Saison S. cerevisiae
9
Wine Renaissance_Vivace S.cerevisiae bayanus
10
Wine Collection_CépagePinot® S. cerevisiae
11
Beer Belgian_Wit S. cerevisiae
12
Beer Saflager_S23 S. cerevisiae
13
Beer Verdant_IPA S. cerevisiae
14
Beer NWS_Ale S. cerevisiae
15
Beer LalBrew_Köln S. cerevisiae
16
Beer BRY97_American S. cerevisiae
17
Wine Viniflora®_PRELUDE™ Torulaspora delbruecki
Wine Viniflora®_CONCERTO™ Kluyveromyces thermotolerans
Beer Philly_Sour Lachancea spp.

(**A**)

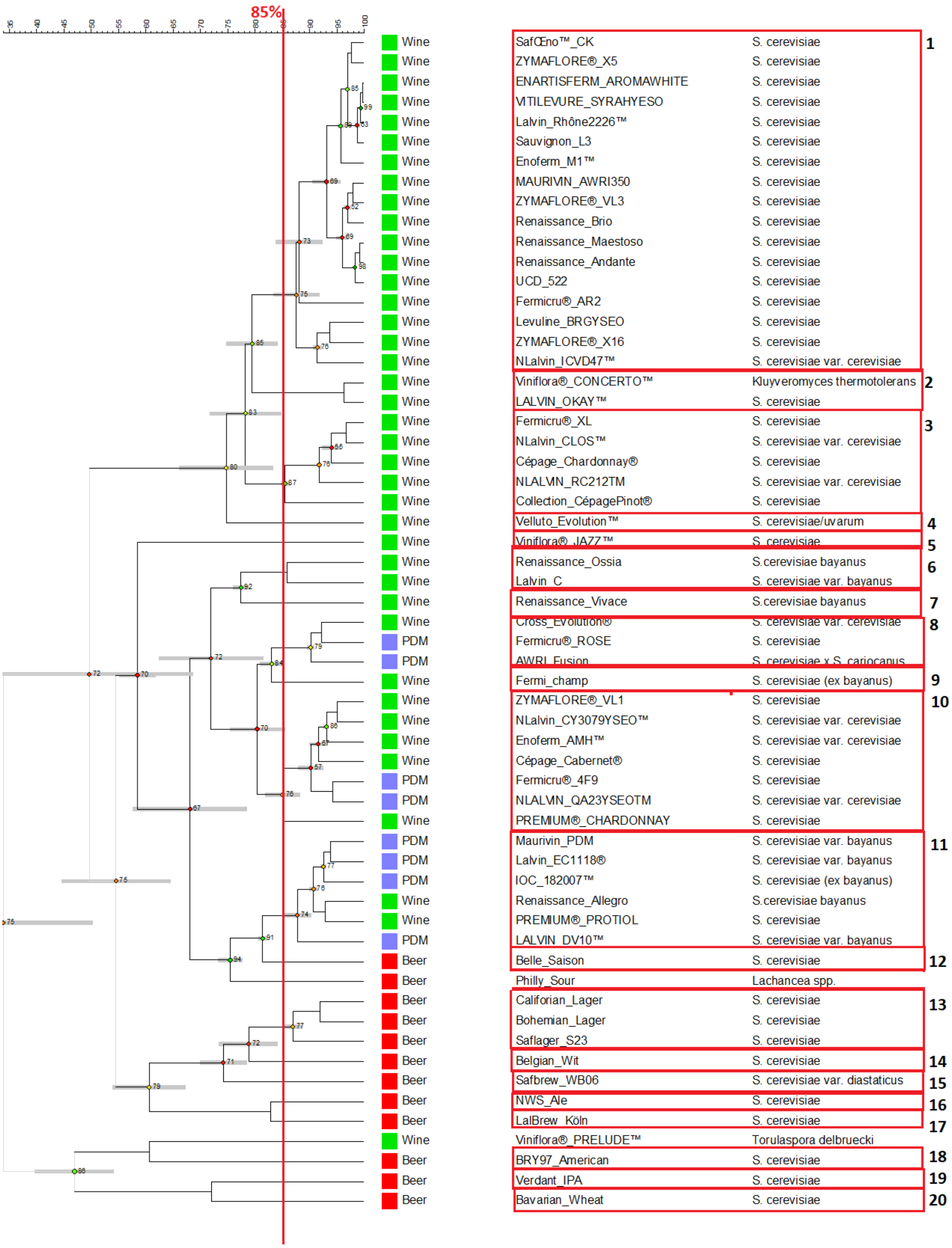
85%
SafŒno™_CK S. cerevisiae
ZYMAFLORE®_X5 S. cerevisiae
ENARTISFERM_AROMAWHITE S. cerevisiae
VITILEVURE_SYRAHYESO S. cerevisiae
Lalvin_Rhône2226™ S. cerevisiae
Sauvignon_L3 S. cerevisiae
Enoferm_M1™ S. cerevisiae
MAURIVIN_AWRI350 S. cerevisiae
ZYMAFLORE®_VL3 S. cerevisiae
Renaissance_Brio S. cerevisiae
Renaissance_Maestoso S. cerevisiae
Renaissance_Andante S. cerevisiae
UCD_522 S. cerevisiae
Fermicru®_AR2 S. cerevisiae
Levuline_BRGYSEO S. cerevisiae
ZYMAFLORE®_X16 S. cerevisiae
NLalvin_ICVD47™ S. cerevisiae var. cerevisiae
1
Viniflora®_CONCERTO™ Kluyveromyces thermotolerans
LALVIN_OKAY™ S. cerevisiae
2
Fermicru®_XL S. cerevisiae
NLalvin_CLOS™ S. cerevisiae var. cerevisiae
Cépage_Chardonnay® S. cerevisiae
NLALVIN_RC212TM S. cerevisiae var. cerevisiae
Collection_CépagePinot® S. cerevisiae
3
Velluto_Evolution™ S. cerevisiae/uvarum
4
Viniflora®_JAZZ™ S. cerevisiae
5
Renaissance_Ossia S.cerevisiae bayanus
Lalvin_C S. cerevisiae var. bayanus
6
Renaissance_Vivace S.cerevisiae bayanus
7
Cross_Evolution® S. cerevisiae var. cerevisiae
Fermicru®_ROSE S. cerevisiae
AWRI_Fusion S. cerevisiae x S. cariocanus
8
Fermi_champ S. cerevisiae (ex bayanus)
9
ZYMAFLORE®_VL1 S. cerevisiae
NLalvin_CY3079YSEO™ S. cerevisiae var. cerevisiae
Enoferm_AMH™ S. cerevisiae var. cerevisiae
Cépage_Cabernet® S. cerevisiae
Fermicru®_4F9 S. cerevisiae
NLALVIN_QA23YSEOTM S. cerevisiae var. cerevisiae
PREMIUM®_CHARDONNAY S. cerevisiae
10
Maurivin_PDM S. cerevisiae var. bayanus
Lalvin_EC1118® S. cerevisiae var. bayanus
IOC_182007™ S. cerevisiae (ex bayanus)
Renaissance_Allegro S.cerevisiae bayanus
PREMIUM®_PROTIOL S. cerevisiae
LALVIN_DV10™ S. cerevisiae var. bayanus
11
Belle_Saison S. cerevisiae
12
Philly_Sour Lachancea spp.
Califorian_Lager S. cerevisiae
Bohemian_Lager S. cerevisiae
Saflager_S23 S. cerevisiae
13
Belgian_Wit S. cerevisiae
14
Safbrew_WB06 S. cerevisiae var. diastaticus
15
NWS_Ale S. cerevisiae
16
LalBrew_Köln S. cerevisiae
17
Viniflora®_PRELUDE™ Torulaspora delbruecki
BRY97_American S. cerevisiae
18
Verdant_IPA S. cerevisiae
19
Bavarian_Wheat S. cerevisiae
20
Wine
PDM
Beer

(**B**)

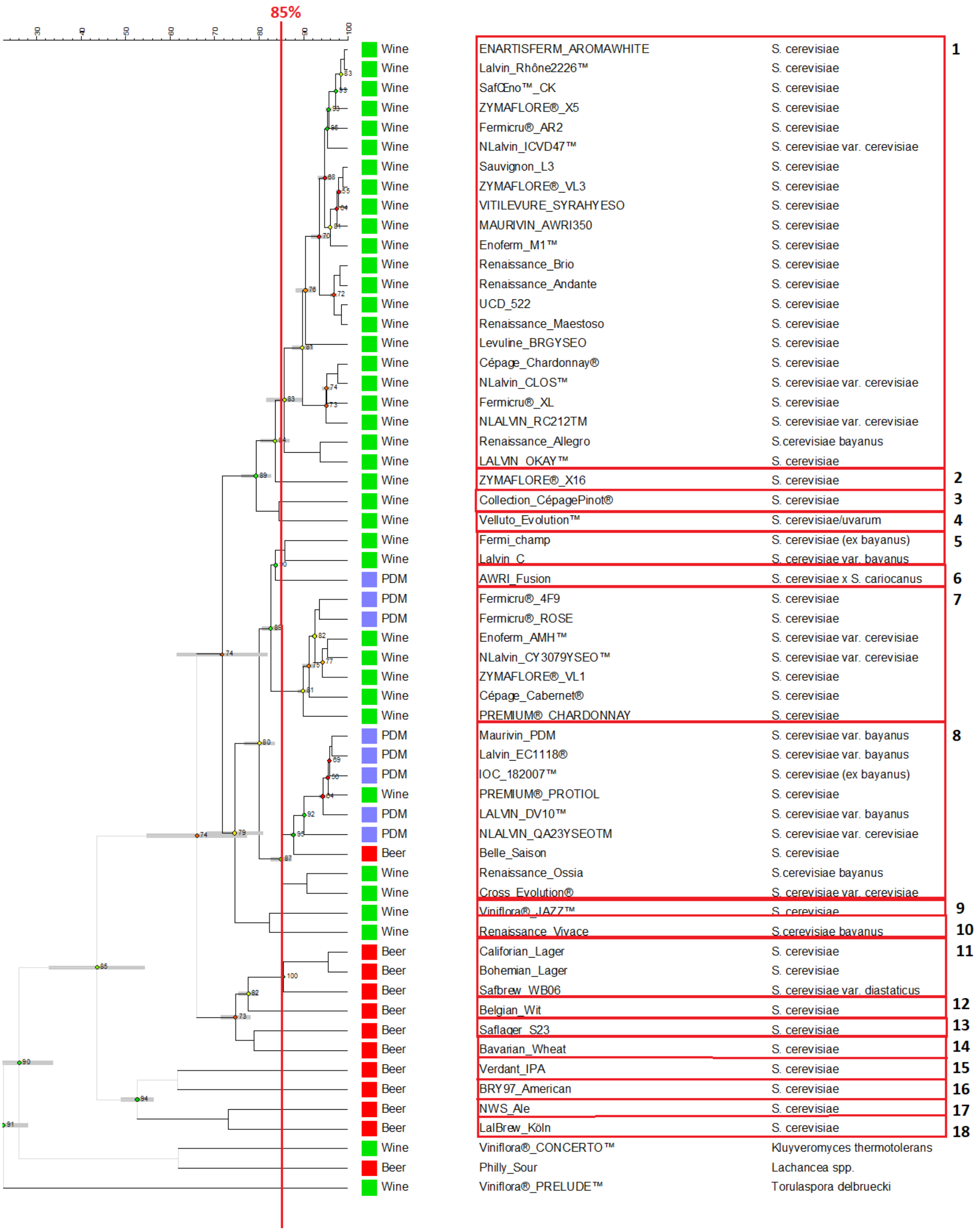

(C)

Figure S3. Cluster analysis of high mass profiles of 59 commercial strains (47 wine and 12 brewing strains) grown on YPD agar (**A**) High Mass, (**B**) Low Mass and (**C**) High & Low Combined.

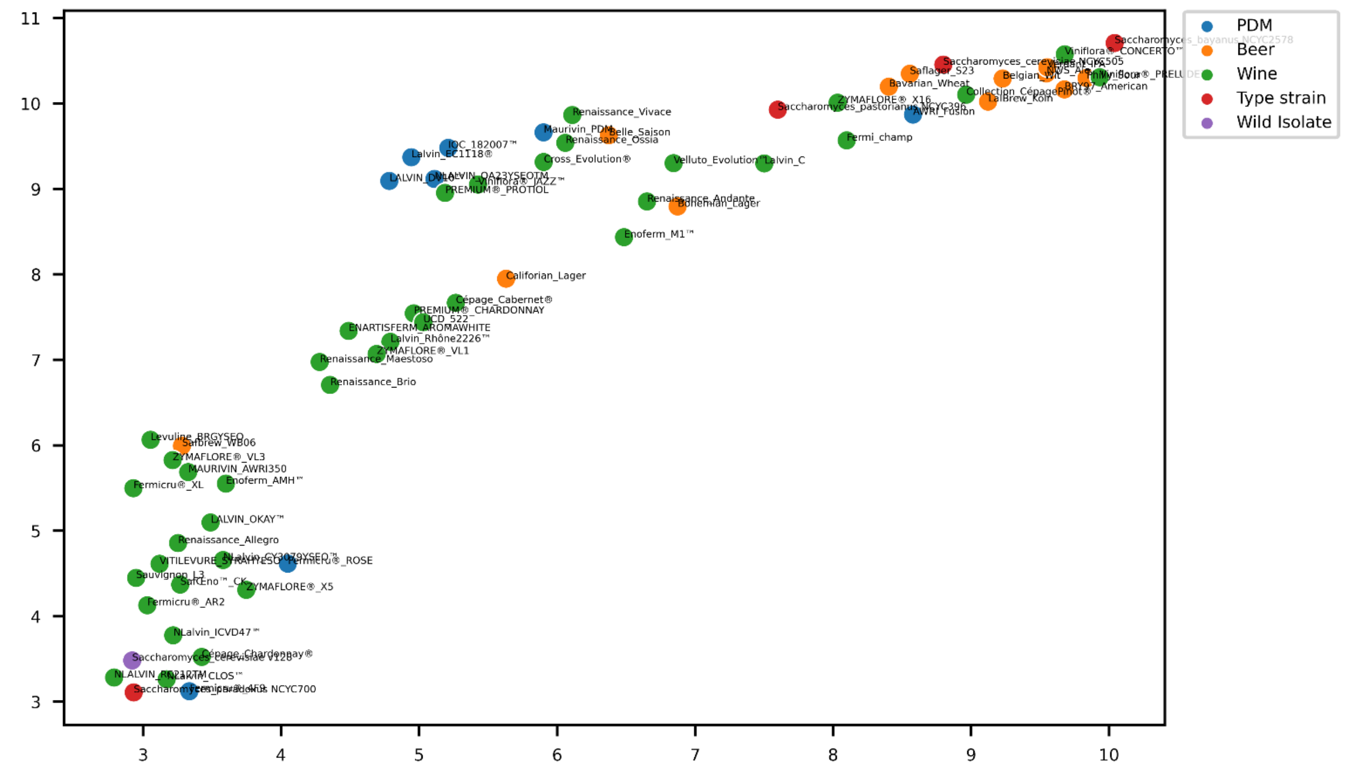
PDM
Beer
Wine
Type strain
Wild Isolate
11
10
9
8
7
6
5
4
3
3
4
5
6
7
8
9
10

(**A**)

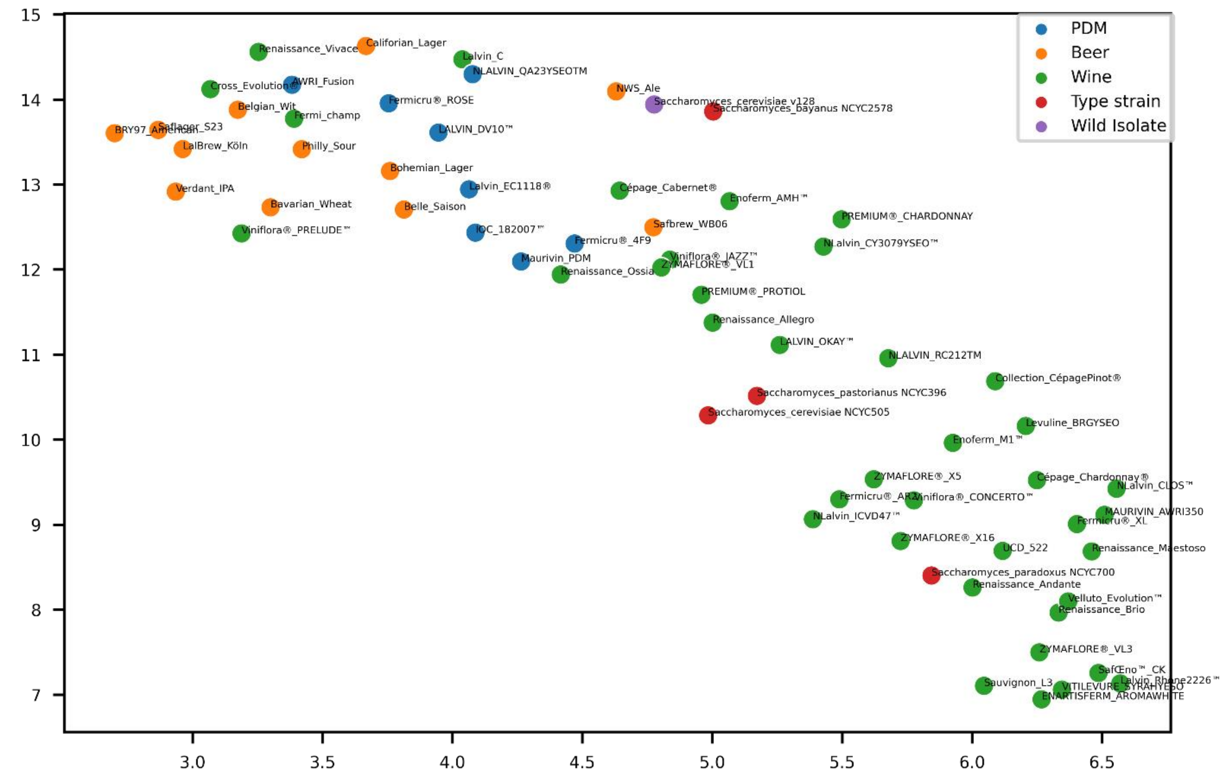
PDM
Beer
Wine
Type strain
Wild Isolate
15
14
13
12
11
10
9
8
7
3.0
3.5
4.0
4.5
5.0
5.5
6.0
6.5

(**B**)

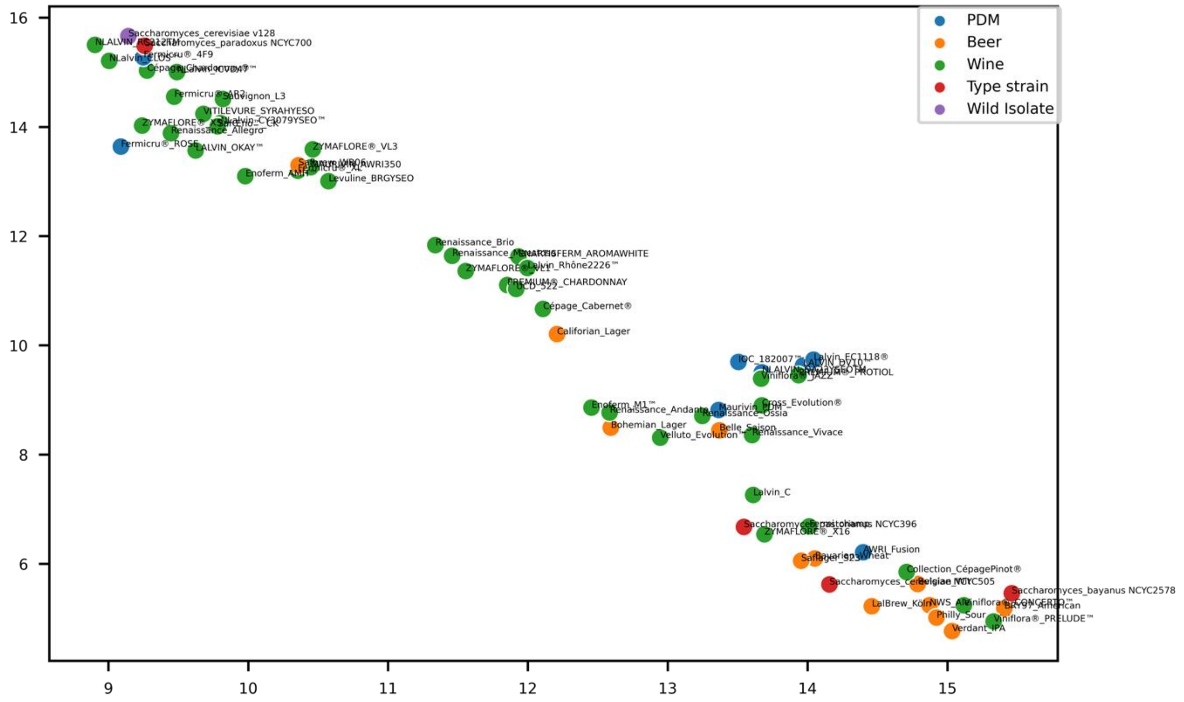

(C)

Figure S4. UMAP analysis of (**A**) high mass, (**B**) low mass and (**C**) high & low combined data of 62 yeast strains-45 wine strains (green/purple), 12 brewing strains (red), *S. cerevisiae* v128 (blue), *S. cerevisiae* NYC 505 (yellow), *S. paradoxus* NCYC 700(yellow), *S. pastorianus* NCYC 396 (yellow), *S. bayanus* NCYC 2578 (yellow).

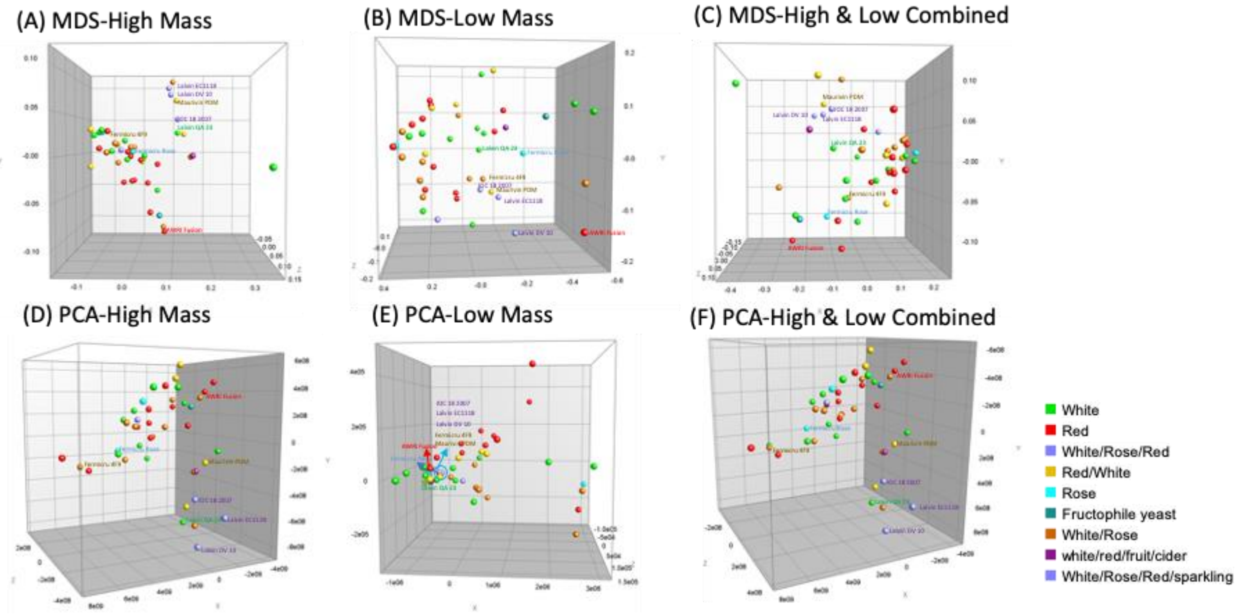

Figure S5. MDS and PCA analysis of 45 commercial wine *Saccharomyces* strains. (**A**) high mass; (**B**) low mass and (**C**) high and low combined data (**D**) high mass and (**E**) low mass, and (**F**) high and low combined.

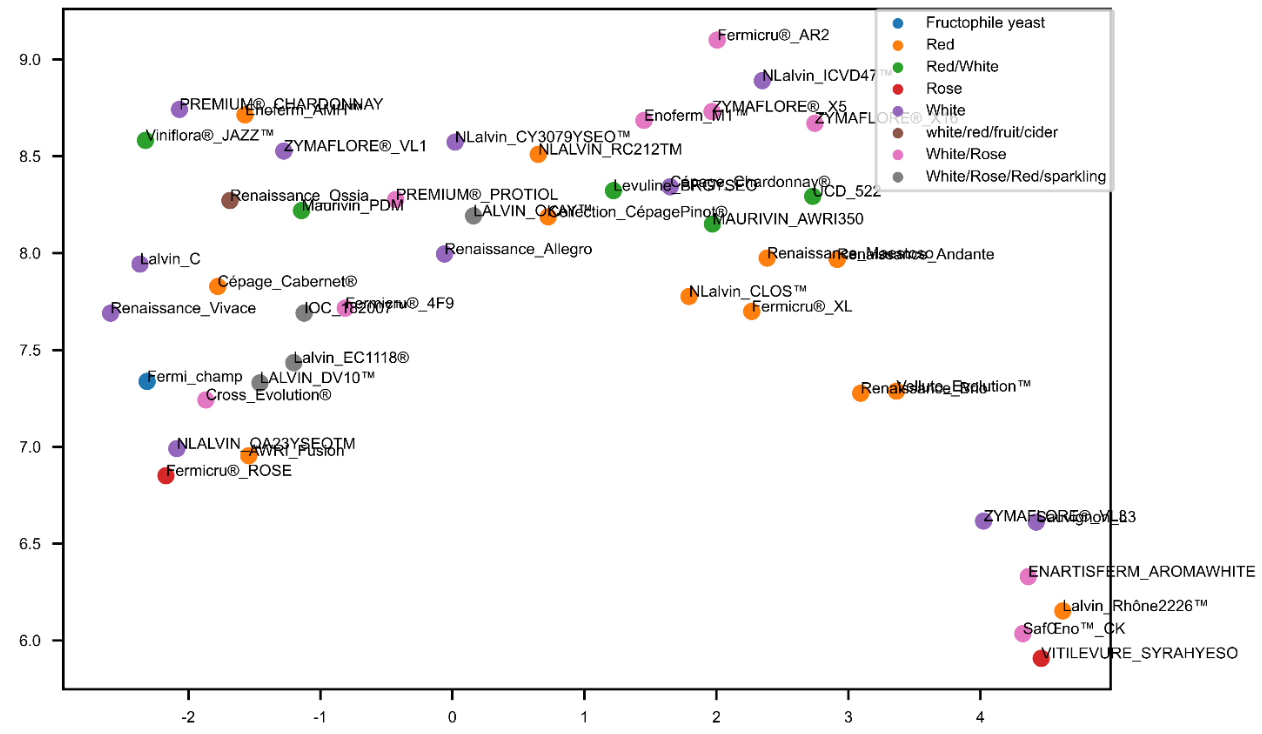

(A)

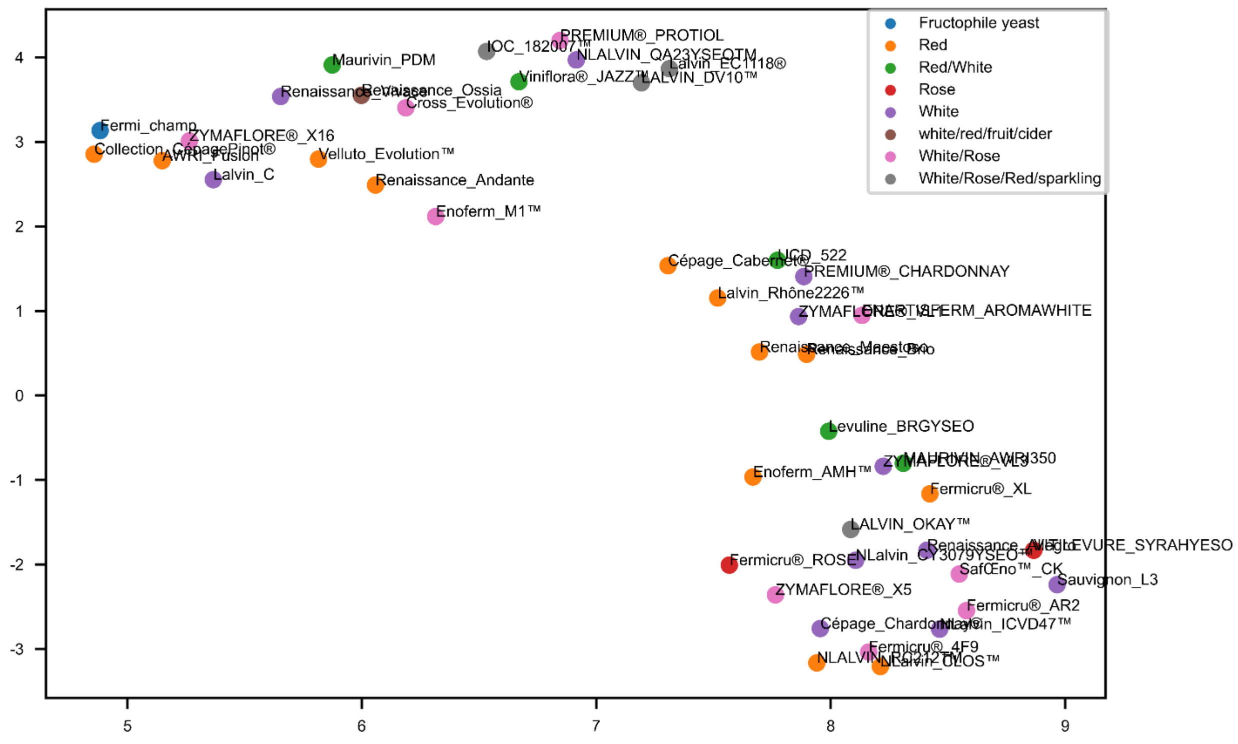

(B)

Figure S6. UMAP analysis of (**A**) Low mass and (**B**) High & Low combined data of 45 commercial wine *Saccharomyces* strains.

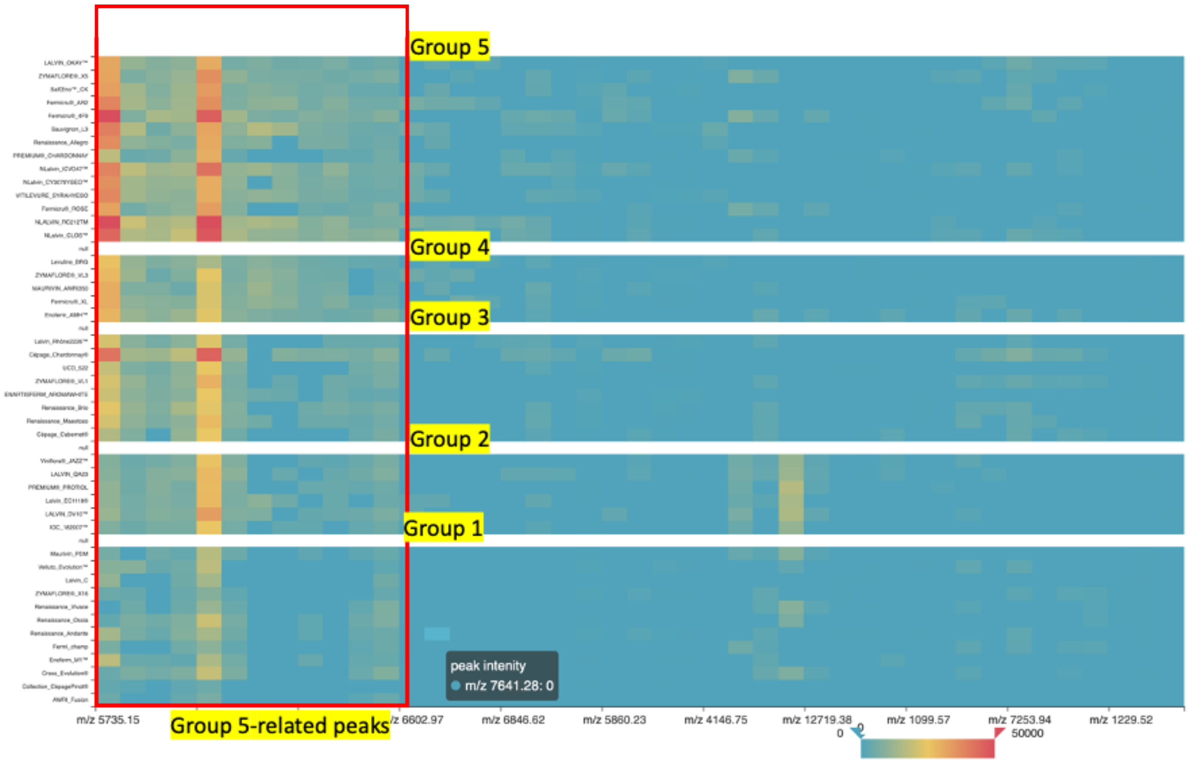

Figure S7. Heatmap of peak classes detected from 45 commercial wine strains and grouped according to UMAP analysis. Red colour represents the highest peak intensity, whereas the blue colour represents the lowest peak intensity.

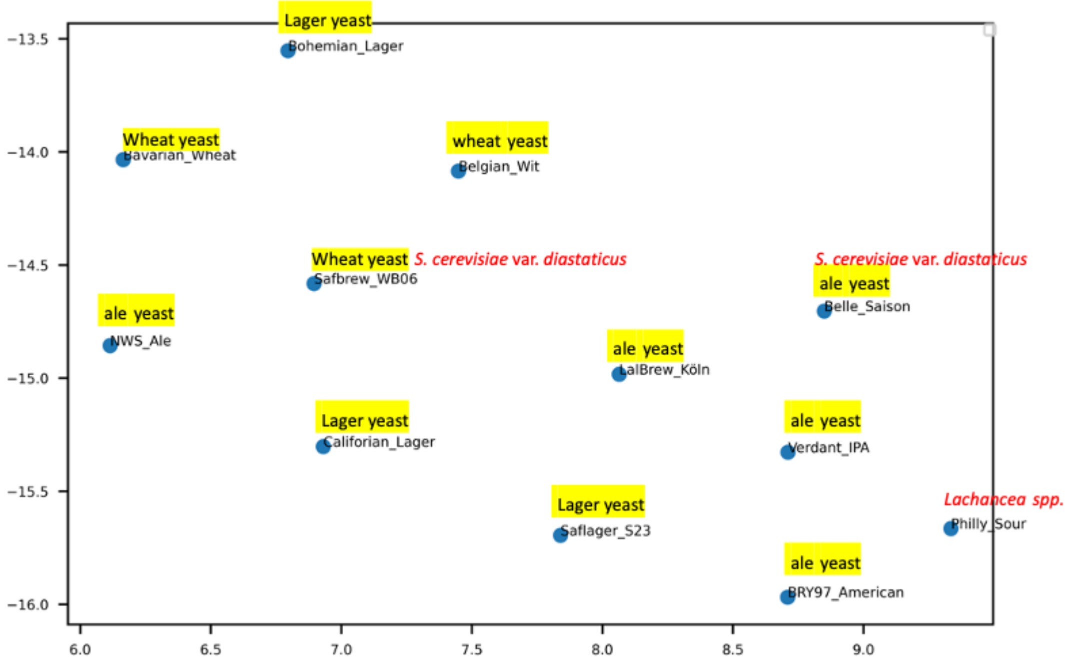

(**A**)

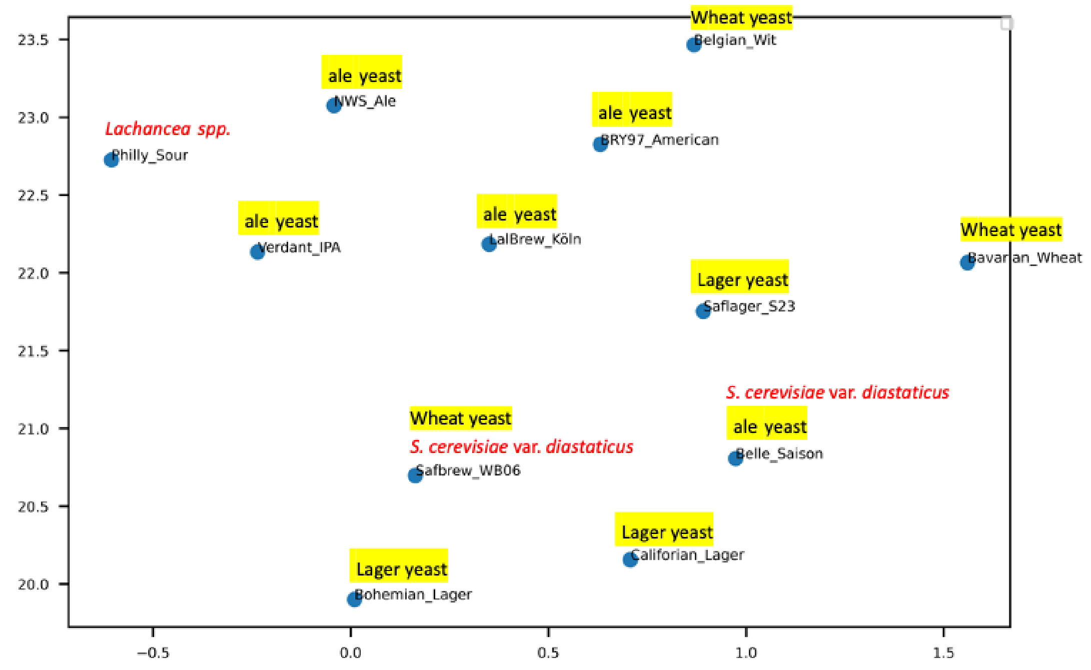

(**B**)

Figure S8. UMAP analysis of (**A**) Low mass and (**B**) High & Low combined data of 12 commercial brewing strains.
